# Supplementary material for: Environmental conditions associated with initial northern expansion of anatomically modern humans
Source: Nat Commun. 2024 May 22;15:4364. doi: 10.1038/s41467-024-48762-8 (PMC11111671; doi:10.1038/s41467-024-48762-8)
Supplement: Supplementary file 1 — Supplementary Information [file 41467_2024_48762_MOESM1_ESM.pdf]

Environmental conditions associated with initial northern expansion of anatomically modern humans

Frédérik Saltré, Joël Chadœuf, Thomas Higham, Monty Ochocki, Sebastián Block, Ellyse Bunney, Bastien Llamas, Corey J. A. Bradshaw

Supplementary Tables

Supplementary Table 1 – Correspondence between biome types simulated by BIOME4 (each number indicates the biome type code in the model) or reconstructed based on fossil pollen and plant-macrofossil data (BIOME6000) and *forest* versus *grassland* categories.

**BIOME4/BIOME6000 plant functional types**	**Type of vegetation (forest/grassland)**
(1) Tropical Evergreen Forest Water; (2) Tropical semi-deciduous forest; (3) Tropical Deciduous Forest/Woodland; (4) Temperate Deciduous Forest; (5) Temperate Conifer Forest; (6) Warm Mixed Forest; (7) Cool Mixed Forest; (8) Cool Conifer Forest; (9) Cold Mixed Forest, (10) Evergreen Taiga, Montane Woodlands; (11) Deciduous Taiga, Montane Forest, (15) Temperate Schlerophyll Savanna, (17) Open Conifer Woodland	forest
(12) Temperate Xerophytic Shrubland; (13) Tropical Xerophytic Shrubland; (14) Tropical Savanna; (16) Temperate Broadleaved Savanna; (18) Boreal Parkland; (19) Tropical Grassland; (20) Temperate Grassland; (21) Desert; (22) Steppe Tundra; (23) Shrub Tundra; (24) Dwarf Shrub Tundra; (25) Prostrate Shrub Tundra; (26) Cushion Forb Lichen Moss tundra; (27) Barren; (28) Ice land	grassland

Supplementary Table 2 – Length and median travel speed trajectories (and confidence interval estimated as the 25 and 75th percentile of the speed of each grid cell along a given trajectory) between seven pairs of source-destination locations (identified as brown-yellow markers in each map of Supplementary Figure 5). The 'optimal' and 'sub-optimal' trajectories between a source and its related destination are highlighted in blue and orange (following the colour coding of Supplementary Figure 5). Source data are provided as a Source Data file

Source-destinations	Path ID	Path length (km)	Travel speed (km yr^{-1})
Fertile Crescent & Beringia	1	9,612,924	71.77 (19.49 – 290.73)
	2	10,588,221	99.38 (22.15 – 448.53)
	3	9,062,715	82.35 (15.09 – 430.04)
	4	8,746,212	69.24 (15.37 – 215.63)
	5	9,061,183	60.04 (23.73 – 346.93)
	6	12,974,904	53.06 (11.71 – 185.36)
Fertile Crescent & Scandinavia	1	2,678,125	3.19 (0.49 – 20.62)
	2	2,695,042	4.54 (0.78 – 10.95)
	3	4,058,669	13.42 (3.23 – 45.70)
	4	5,306,830	16.63 (5.50 – 36.46)
	5	7,897,455	73.92 (16.33 – 236.34)
	6	8,686,522	93.11 (26.08 – 238.48)
	7	5,075,781	19.88 (5.19 – 63.10)
Fertile Crescent & Japan	1	7,721,195	73.05 (13.53 – 256.50)
	2	8,967,363	76.45 (17.31 – 275.06)
	3	10,340,752	103.30 (30.48 – 343.98)
	4	11,196,138	81.05 (19.52 – 292.22)
	5	7,996,580	60.82 (10.04 – 197.06)
	6	11,638,832	68.45 (12.45 – 250.05)
	7	12,275,345	38.8 (7.34 – 248.79)
	8	7,842,338	96.8 (27.69 – 474.32)
Fertile Crescent & Portugal	1	3,149,024	2.77 (1.33 – 14.74)
	2	4,463,796	7.4 (2.11 – 27.56)
	3	4,999,744	7.51 (2.12 – 34.43)
	4	6,740,019	33.94 (7.54 – 103.86)
	5	7,632,951	34.74 (7.05 – 123.78)
	6	7,403,780	28.28 (5.73 – 116.56)
Beringia & Central America	1	3,867,094	12.04 (1.65 – 48.37)
	2	5,237,561	8.09 (1.68 – 39.91)
	3	6,600,352	11.51 (2.99 – 42.31)
	4	5,492,430	25.56 (4.93 – 81.78)
	5	7,011,209	9.29 (2.14 – 42.84)
	6	5,664,231	10.6 (1.57 – 68.99)
	7	4,215,099	15.41 (2.84 – 74.85)
	8	3,866,951	6.53 (1.28 – 38.03)
Central & Eastern Brazil	1	734,145	51.7 (18.31 – 231.61)
	2	1,028,451	54.13 (18.98 – 210.67)
	3	1,957,848	119.59 (31.09 – 295.56)
	4	1,030,956	35.59 (18.35 – 119.23)
	5	1,020,389	31.4 (8.72 – 96.86)
	6	831,273	44.64 (10.57 – 152.55)
Central America & Chile	1	1,314,137	92.56 (19.13 – 286.81)
	2	1,369,241	32.22 (7.19 – 236.99)
	3	1,532,586	93.71 (8.45 – 386.89)
	4	1,303,426	103.55 (26.09 – 361.88)
	5	1,527,696	56.82 (12.31 – 290.59)
	6	1,235,267	95.71 (9.29 – 226.90)
	7	1,513,954	86.32 (13.67 – 307.04)

Supplementary Figures

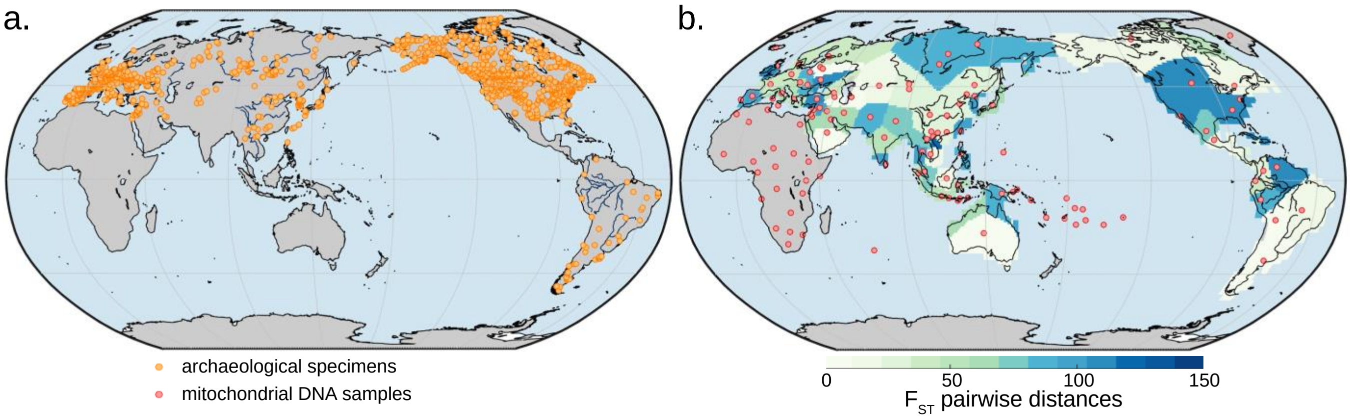

Supplementary Figure 1 – Spatial distribution of **a**. reliable, high-quality (see *Methods*) radiocarbon-dated archaeological specimens indicating human presence (orange dots), and **b**. mitochondrial DNA samples (red dots) used to generate the map of F_{ST} paiwise distances ranging from light green (short F_{ST} paiwise distances) to dark blue (long F_{ST} pairwise distances). The red dots represent the centroids (the size of each centroid is indicated by the colourbar of F_{ST} pairwise distances) of the 96 geographical locations (either country or region/province in countries) used to group the 31 mitochondrial haplogroups compiled form the 67,643 human mitochondrial control region sequences from Genebank.

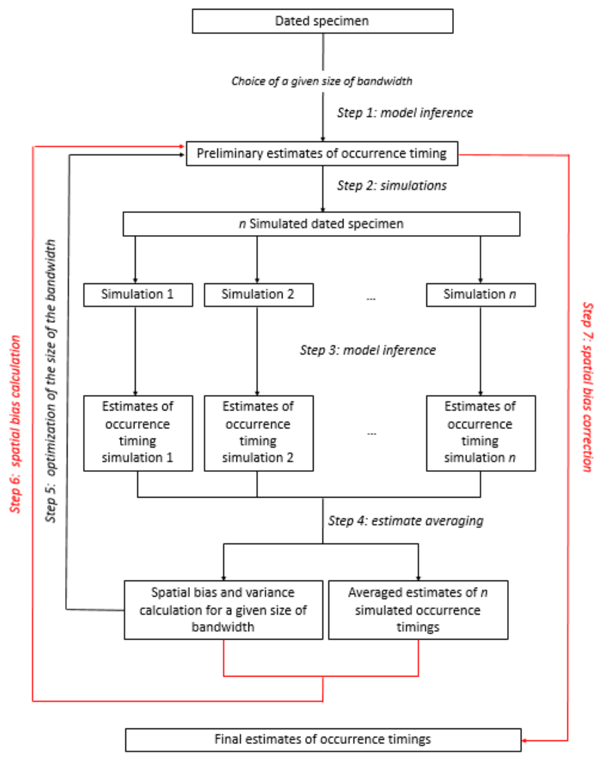

Supplementary Figure 2 – Flow chart of the spatial bias-correction procedure.

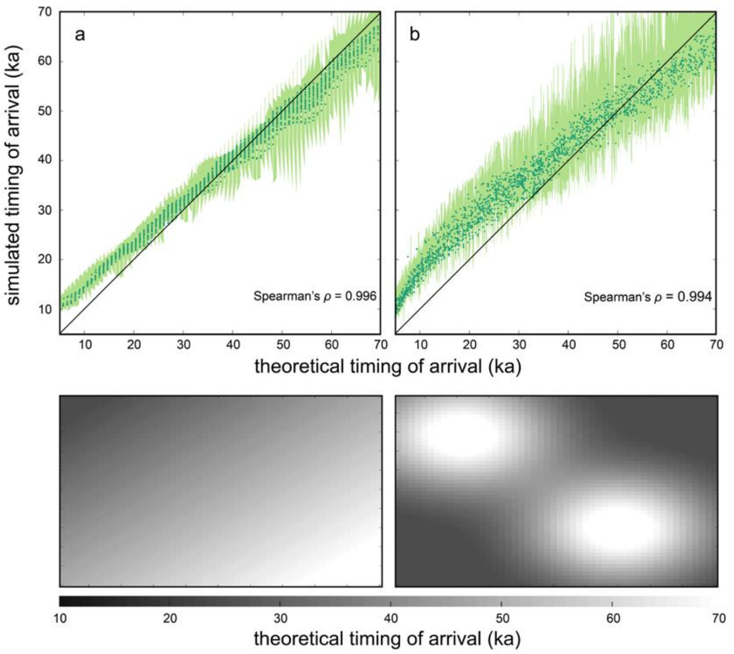

Supplementary Figure 3 – Correlation between observed and estimated regional timing of occurence (1 ka = 1000 years ago) for scenarios based on **a**. gradual peopling of a landscape from a single entrance, and **b.** two entrances in the landscape. Correlations (Spearman's ρ rank correlation coefficient) are calculated over 100 replicates of the pairwise comparisons of the theoretical and inferred timing of occurrence. Bold black lines indicate the median correlation across all repetitions, dark-shaded envelopes represent limits determined by the 25th and 75th percentiles, and light-shaded envelopes indicate the lower and upper limits determined by the 2.5th and 97.5th percentiles. The line in each panel assumes a perfect relationship between observed and inferred values. Bottom panels display the type of expansion pattern for each scenario (left = single entrance; right = two entrances).

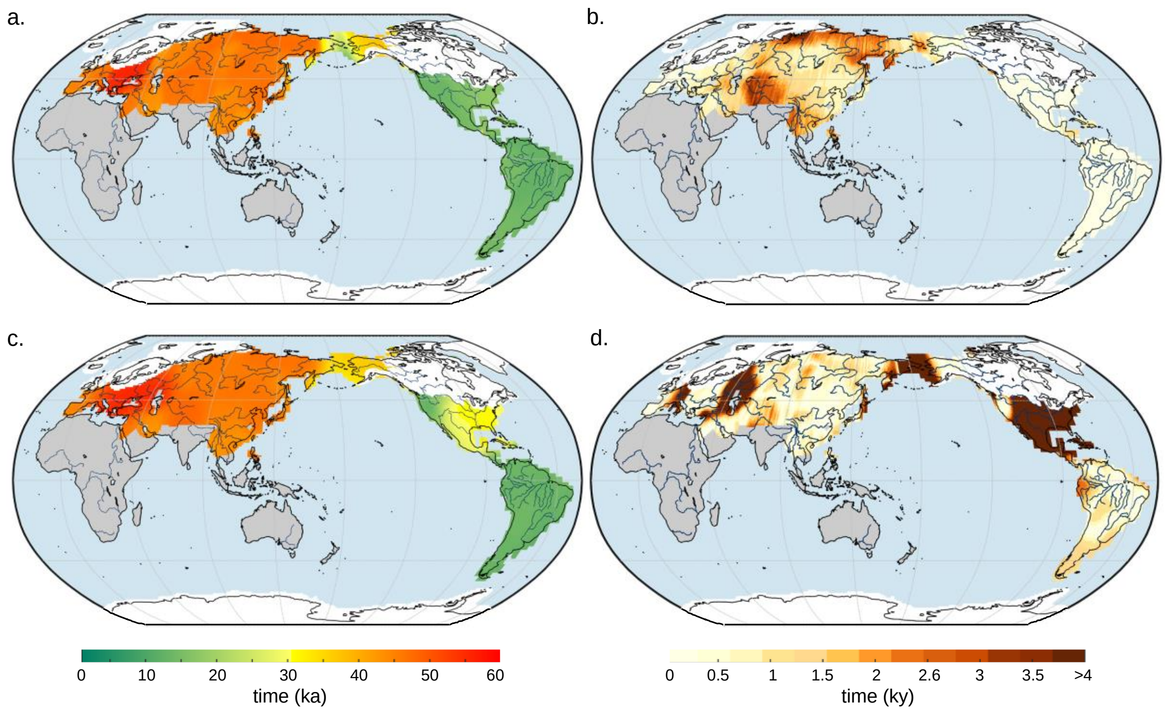

Supplementary Figure 4 – Sensitivity of the spatial pattern of the timing of arrival of the first anatomically modern humans to the quality-rating protocol at a spatial resolution of 1 ° × 1 ° latitude. Maps of estimated timing of arrival of the first anatomically modern humans (1 ka = 1000 years ago) estimated from **a**. high-quality ages of archaeological specimens along with **b**. its confidence interval and **c**. based in all available data (i.e., without any quality rating; see details in *Methods*). **d**. Maps of time differences (i.e., absolute value for each grid cell; 1 ky = 1000 years) between estimated data in **a** and **c**. Also shown are major rivers (dark blue), areas with no estimates (grey), and the ice-sheet coverage (white) 16,000 years ago, before humans entered the Americas (see justifications in the main text).

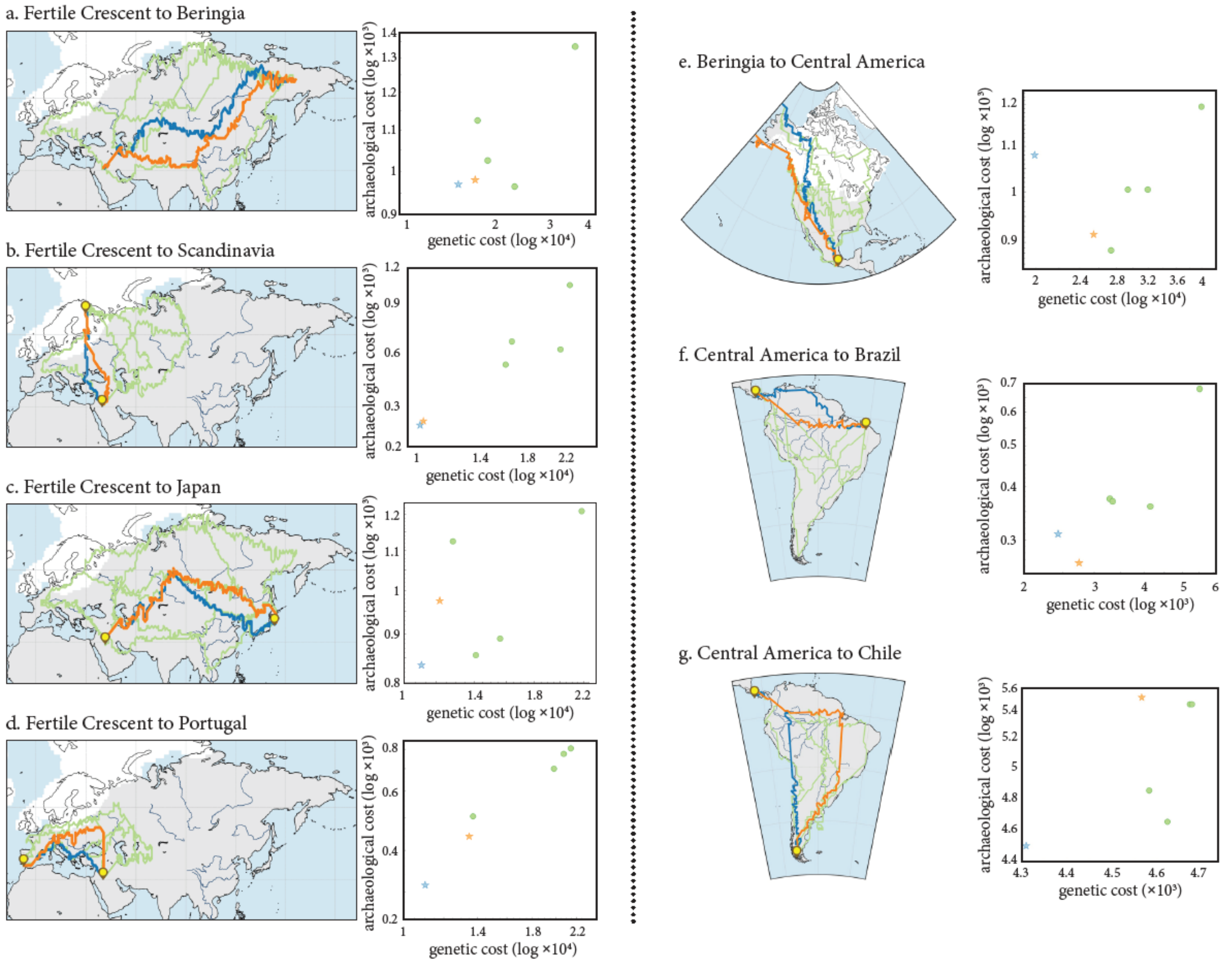

Supplementary Figure 5 – Evaluated trajectories between seven pairs of source-destination locations (identified as brown-yellow markers in each map): **a**. Fertile Crescent and Beringia, **b**. Fertile Crescent and Scandinavia, **c**. Fertile Crescent and Japan, **d**. Fertile Crescent and Portugal, **e**. Beringia and Central America, **f**. Central and Eastern Brazil, **g**. Central America and Chile. For each area, the map is displayed with all trajectories (left panel) along with the scatter plot of the genetic cost of each trajectory as a function of the respective archaeological cost (right panel). Trajectories are ranked by calculating the Euclidean distance between the archaeological and genetic costs and the origin (0,0). The 'optimal' trajectory between a source and its related destination (in blue on each map) represents the shortest Euclidean distance, whereas other non-optimal (but plausible) scenarios are indicated in green. If any other trajectory has a Euclidean distance of the same order of magnitude as the shortest one, this trajectory is also selected as 'optimal' (in orange). Also shown are major rivers (dark blue, see details in *Methods*), and the ice-sheet coverage (white) 16,000 years ago, before humans entered the Americas (see justifications in the main text). Source data are provided as a Source Data file

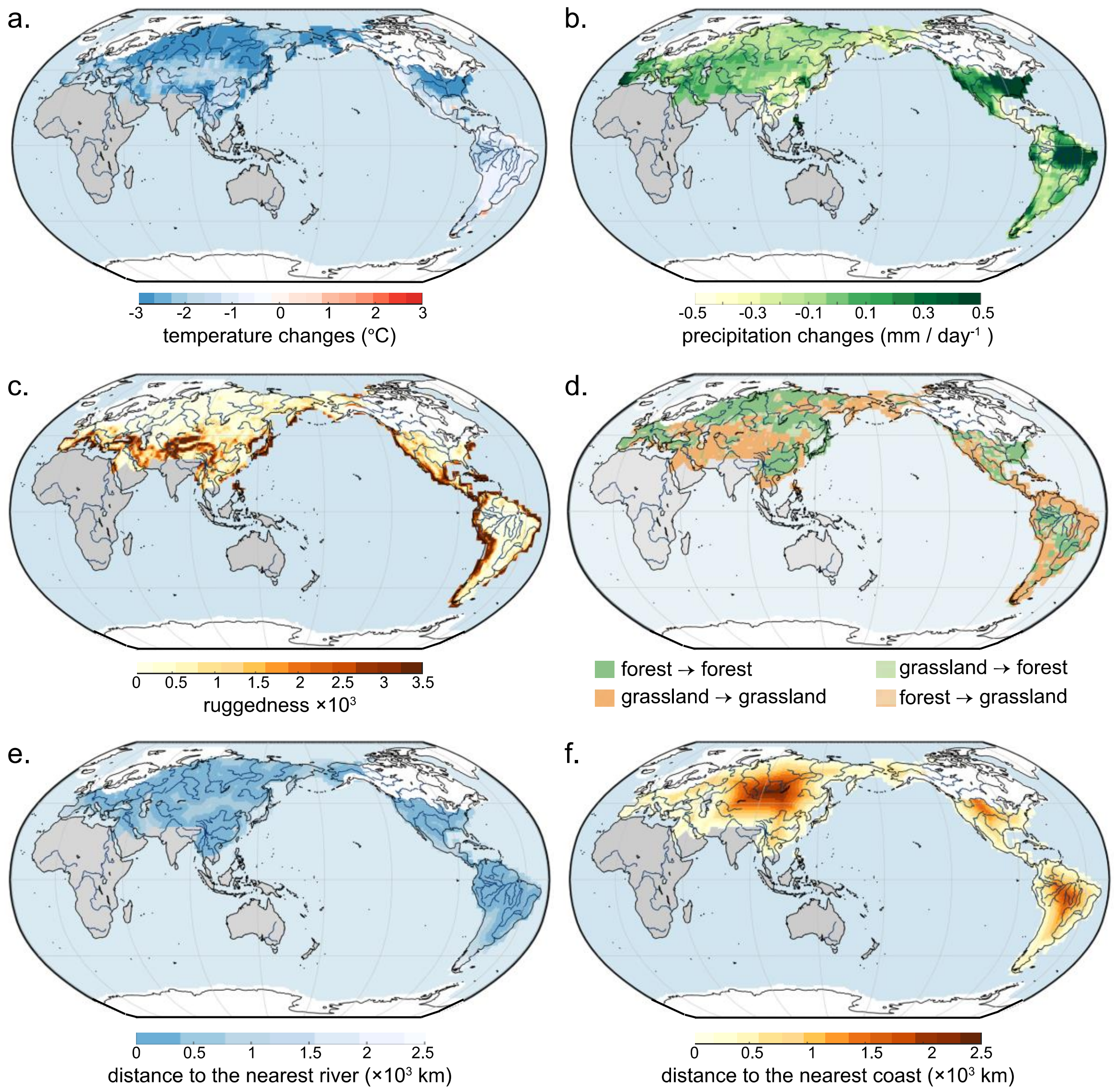

Supplementary Figure 6 – Environmental variables used to explain the peopling of Eurasia and the Americas at a spatial resolution of 1 ° × 1° latitude. **a**. Mean annual temperature anomalies and **b**. mean annual precipitation anomalies at the estimated timing of human arrival in a grid cell (Supplementary Figure 4a) relative to 90 ka (see justification in *Methods*). Both mean annual temperature and precipitation were simulated using the HadCM3 Atmosphere-Ocean General Circulation Model. **c**. Present-day topographic ruggedness index (landscape accessibility) calculated as the difference in elevation between a given cell and its 8 neighbouring central cells, based on the the ETOPO1 global relief model of the Earth's surface (see details in *Methods*). **d**. Changes in dominant vegetation type (forest or grassland biome) simulated by the BIOME4 model (see details in *Supplementary Method 2*) at the estimated timing of arrival in each grid cell (Supplementary Figure 4a) and relative to 90 ka. **e**. distance to nearest major river calculated as the shortest Euclidean distance of each grid cell to a grid cell including one of these rivers. **f**. distance to the nearest coast calculated as the shortest Euclidean distance of each grid cell to a grid cell at the estimated timing of human arrival in a grid cell and with a limit of the land mass accounting for the change in sea level during the Late Pleistocene (see details in *Methods*). Also shown are major rivers (dark blue; see details in *Methods*), areas with no estimates (grey) and the ice-sheet coverage (white) 16,000 years ago, before humans entered the Americas (see justifications in the main text).

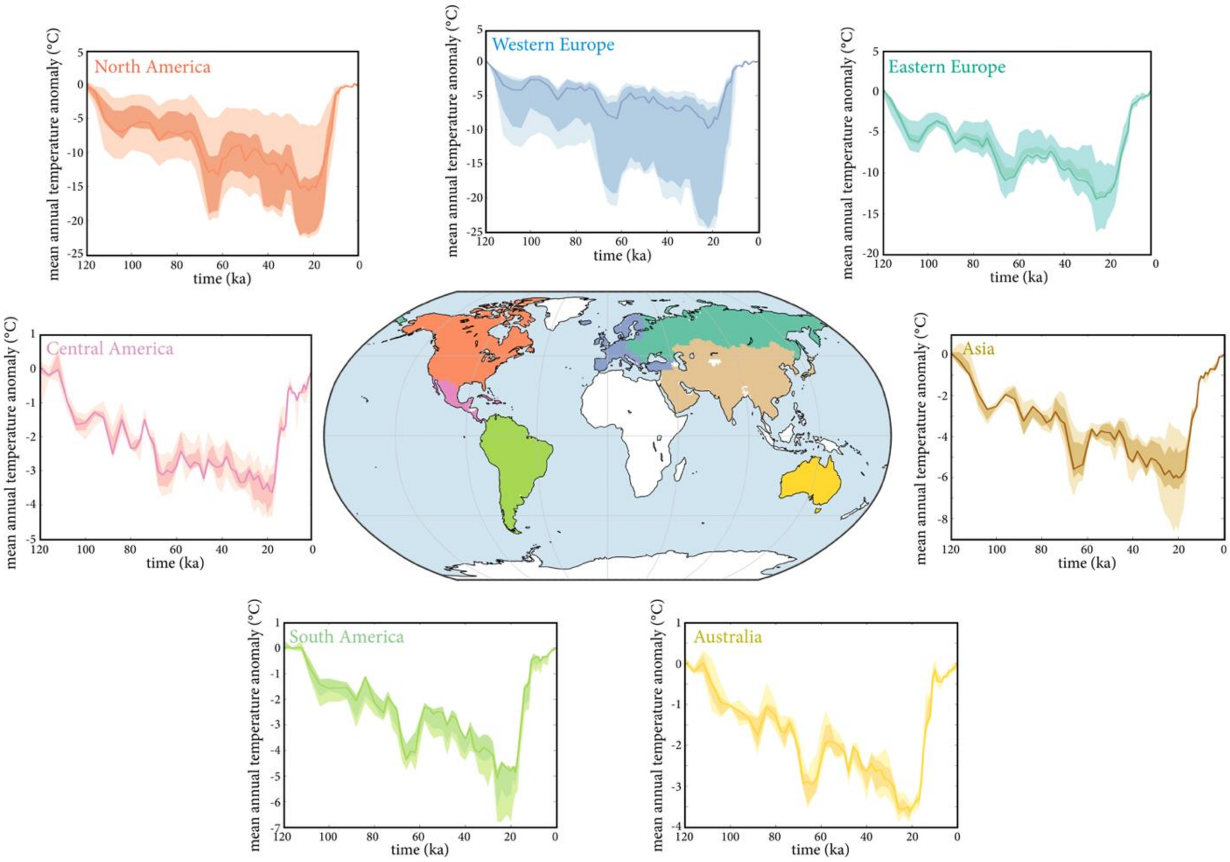

Supplementary Figure 7 – Changes in mean annual temperature anomalies over the last 120 ka (ka = 1000 years) relative to the present (baseline centred on 1950 AD, anomaly) simulated from the HadCM3 Atmosphere-Ocean General Circulation Model (see details in *Methods*). The magnitude of change in mean annual temperature is calculated every millennium for North America (red), Central America (pink), South America (light green), Western Europe (blue), Eastern Europe (dark green), Asia (brown), and Australia (yellow). Plots show the median value (solid line), the 25th and 75th percentiles (dark shading) and the 2.5th and 97.5th percentiles (light shading) across each region. The temporal resolution of the data is 1-k slices back to 22 ka, 2-k from 22 to 80 ka, and 4-k to 120 ka at a 1 º × 1 º spatial resolution. Also shown are the areas that we ignored because of insufficient data (white; see main text). Source data are provided as a Source Data file.

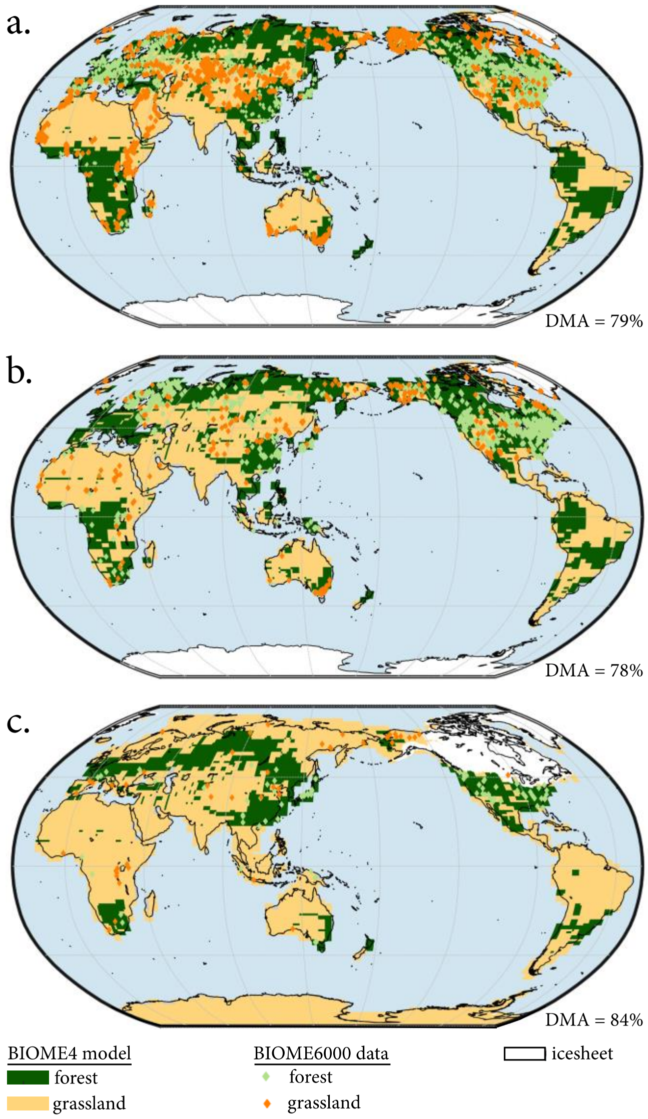

Supplementary Figure 8 – Reconstructed vegetation types (defined as *forest* or *grassland*) from fossil pollen and plant-macrofossil data, extracted from the BIOME6000 database Version 4.2 (bridge.bris.ac.uk/resources/Databases/BIOMES_data) superimposed on BIOME4 simulated vegetation types (also classified as *forest* or *grassland*) using HadCM3 climates (see details in *Supplementary Method 2*) for **a**. the present, **b**. 6 ka, and **c**. 18 ka. For each time period, the data-model agreement (DMA) statistic indicates the percentage grid cell where the vegetation simulated using BIOME4 agrees with BIOME6000 data. Also shown in white is the extent of the ice sheet for each time period.

Supplementary Discussion

Uncertainties in the estimated patterns of human expansion

We reconstructed the geographic patterns of the timing of human expansion (Supplementary Figure 4a) following the major exit out of Africa > 65 ka[1,2] by applying a comprehensive, spatio-temporal method[3] to the high-quality (i.e., suitable dates; see quality-rating approach in *Methods*) archaeological records scattered across Eurasia and the Americas (Supplementary Figure 1a). This statistical method corrects for both inherent taphonomic and spatial heterogeneity biases in the archaeological records, and returns for each 1 ° × 1 ° terrestrial grid cell an average (± standard deviation) estimate of the timing of arrival (see details, sensitivity analysis, and validation in *Supplementary Method*)[3]. Assuming a starting point of expansion from the Fertile Crescent, we estimated that humans spread rapidly across eastern Europe and Asia by 48–47 ka to reach Scandinavia through the Caucasus, and later eastern Asia (by 45–44 ka) and Beringia (~ 34 ka)[4,5] via a south-eastern path to the Caspian Sea, and northern Mongolia. At approximately the same time, westward human migration reached the Iberian coast by 44 ka via the Mediterranean coast. The entrance to the Americas first occurred after a ~ 18.4-ky migration hiatus in Beringia via the Pacific Northwest coast[6], followed by a second route ~ 2.5 ky later through a newly formed, ice-free corridor inland[7,8]. Humans subsequently reached South America and spread following both clockwise and anti-clockwise directions no later than 14 ka[9,11,12].

Supplementary References

1 Mellars, P. Why did modern human populations disperse from Africa ca. 60,000 years ago? A new model. *Proceedings of the National Academy of Sciences* **103**, 9381-9386 (2006). https://doi.org:10.1073/pnas.0510792103

2 Groucutt, H. S. *et al.* Rethinking the dispersal of Homo sapiens out of Africa. *Evolutionary Anthropology: Issues, News, and Reviews* **24**, 149-164 (2015). https://doi.org:10.1002/evan.21455

3 Saltré, F. *et al.* Climate-human interaction associated with southeast Australian megafauna extinction patterns. *Nat. Commun.* **10**, 5311 (2019). https://doi.org:10.1038/s41467-019-13277-0

4 Kuzmin, Y. V., Kosintsev, P. A., Razhev, D. I. & Hodgins, G. W. L. The oldest directly-dated human remains in Siberia: AMS 14C age of talus bone from the Baigara locality, West Siberian Plain. *Journal of Human Evolution* **57**, 91-95 (2009). https://doi.org:10.1016/j.jhevol.2009.04.003

5 Pitulko, V. V. *et al.* The Yana RHS Site: Humans in the Arctic Before the Last Glacial Maximum. *Science* **303**, 52-56 (2004). https://doi.org:10.1126/science.1085219

6 Llamas, B. *et al.* Ancient mitochondrial DNA provides high-resolution time scale of the peopling of the Americas. *Science Advances* **2** (2016). https://doi.org:10.1126/sciadv.1501385

7 Tamm, E. *et al.* Beringian Standstill and Spread of Native American Founders. *PLOS ONE* **2**, e829 (2007). https://doi.org:10.1371/journal.pone.0000829

8 Hoffecker, J. F., Elias, S. A., O'Rourke, D. H., Scott, G. R. & Bigelow, N. H. Beringia and the global dispersal of modern humans. *Evolutionary Anthropology: Issues, News, and Reviews* **25**, 64-78 (2016). https://doi.org:10.1002/evan.21478

9 Bonatto, S. L. & Salzano, F. M. A single and early migration for the peopling of the Americas supported by mitochondrial DNA sequence data. *Proceedings of the National Academy of Sciences of the United States of America* **94**, 1866-1871 (1997). https://doi.org:10.1073/pnas.94.5.1866

10 Dillehay, T. D. The late Pleistocene cultures of South America. *Evolutionary Anthropology: Issues, News, and Reviews* **7**, 206-216 (1999). https://doi.org:10.1002/1520-6505

11 Dillehay, T. D. *et al.* New Archaeological Evidence for an Early Human Presence at Monte Verde, Chile. *PLOS ONE* **10**, e0141923 (2015). https://doi.org:10.1371/journal.pone.0141923

12 Goebel, T., Waters, M. R. & O'Rourke, D. H. The Late Pleistocene Dispersal of Modern Humans in the Americas. *Science* **319**, 1497-1502 (2008). https://doi.org:10.1126/science.1153569
