# Supplementary material for: Environmental conditions associated with initial northern expansion of anatomically modern humans
Source: Nat Commun. 2024 May 22;15:4364. doi: 10.1038/s41467-024-48762-8 (PMC11111671; doi:10.1038/s41467-024-48762-8)
Supplement: Supplementary file 3 — Description of Additional Supplementary Files [file 41467_2024_48762_MOESM3_ESM.pdf]

Description of Additional Supplementary Files

File Name: Supplementary Data 1

Description: Dates included in the analysis. For each of the radiocarbon dates, the following information was included: site name, region, ^{14}C age, laboratory code, and full reference.
