# Supplementary material for: Environmental conditions associated with initial northern expansion of anatomically modern humans
Source: Nat Commun. 2024 May 22;15:4364. doi: 10.1038/s41467-024-48762-8 (PMC11111671; doi:10.1038/s41467-024-48762-8)
Supplement: Supplementary file 5 — Reporting Summary [file 41467_2024_48762_MOESM5_ESM.pdf]

nature portfolio

Corresponding author(s): FREDERIK SALTRE

Last updated by author(s): May 4, 2024

Reporting Summary

Nature Portfolio wishes to improve the reproducibility of the work that we publish. This form provides structure for consistency and transparency in reporting. For further information on Nature Portfolio policies, see our Editorial Policies and the Editorial Policy Checklist.

Statistics

For all statistical analyses, confirm that the following items are present in the figure legend, table legend, main text, or Methods section.

n/a	Confirmed	
☐	☒	The exact sample size (n) for each experimental group/condition, given as a discrete number and unit of measurement
☐	☒	A statement on whether measurements were taken from distinct samples or whether the same sample was measured repeatedly
☐	☒	The statistical test(s) used AND whether they are one- or two-sided<br>*Only common tests should be described solely by name; describe more complex techniques in the Methods section.*
☐	☒	A description of all covariates tested
☐	☒	A description of any assumptions or corrections, such as tests of normality and adjustment for multiple comparisons
☐	☒	A full description of the statistical parameters including central tendency (e.g. means) or other basic estimates (e.g. regression coefficient) AND variation (e.g. standard deviation) or associated estimates of uncertainty (e.g. confidence intervals)
☐	☒	For null hypothesis testing, the test statistic (e.g. F, t, r) with confidence intervals, effect sizes, degrees of freedom and P value noted<br>*Give P values as exact values whenever suitable.*
☒	☐	For Bayesian analysis, information on the choice of priors and Markov chain Monte Carlo settings
☒	☐	For hierarchical and complex designs, identification of the appropriate level for tests and full reporting of outcomes
☐	☒	Estimates of effect sizes (e.g. Cohen's d, Pearson's r), indicating how they were calculated

Our web collection on statistics for biologists contains articles on many of the points above.

Software and code

Policy information about availability of computer code

Data collection	The data collected for this study are available in Supporting information (supplementary table S1) and at https://github.com/FredSaltre/HumanGlobalExpansion.
Data analysis	The R and Matlab scripts to analyse our data are custom-made and freely available at https://github.com/FredSaltre/HumanGlobalExpansion.

For manuscripts utilizing custom algorithms or software that are central to the research but not yet described in published literature, software must be made available to editors and reviewers. We strongly encourage code deposition in a community repository (e.g. GitHub). See the Nature Portfolio guidelines for submitting code & software for further information.

Data

Policy information about availability of data

All manuscripts must include a data availability statement. This statement should provide the following information, where applicable:

- Accession codes, unique identifiers, or web links for publicly available datasets
- A description of any restrictions on data availability
- For clinical datasets or third party data, please ensure that the statement adheres to our policy

The authors declare that data supporting the Figure 1 of this study are available within the paper the Supplementary Information (Supplementary Table S1). All data generated and analyzed during this study as well as the raw data values underlying all reported averages in graphs (i.e., Figure 2 and Extended Figures 6) are available at https://github.com/FredSaltre/HumanGlobalExpansion.

Field-specific reporting

Please select the one below that is the best fit for your research. If you are not sure, read the appropriate sections before making your selection.

- [ ] Life sciences
- [ ] Behavioural & social sciences
- [x] Ecological, evolutionary & environmental sciences

For a reference copy of the document with all sections, see nature.com/documents/nr-reporting-summary-flat.pdf

Ecological, evolutionary & environmental sciences study design

All studies must disclose on these points even when the disclosure is negative.

Study description

We developed a new statistical approach that combines both archaeological and genetic data to infer the most-likely initial expansion routes in Eurasia and the Americas. We first generated continuous maps of unbiased timings of human arrival given the inherent rarity of fossil sites by designing and implementing a new statistical approach to infer spatial patterns of the regional timing of initial human appearance across Eurasia and the Americas. This new statistical approach applied a maximum-likelihood method to correct for the Signor-Lipps effect first developed by Solow (Solow et al, 2006) that we adapted for spatial inference of human-appearance patterns. We then combined the archaeologically derived maps with the spatial patterns of genetic difference in human populations based on present-day mitochondrial DNA to calculate a set of most-likely routes of initial peopling across the continents by using a customized pathway algorithm(based on a a Metropolis simulated annealing algorithm, Metrolpolis et al, 1953)

Research sample

We gathered 24,524 radiocarbon-dated archaeological specimens indicating human presence (i.e., human remains, cultural layers or lithic industry, single artefacts, clear hearths, clear butchering, anthropogenic modification, cave art, portable art, living floors, and probable hearths) from four databases: (i) the Radiocarbon Palaeolithic Europe Database (INQUA8: 10,522 ages; ees.kuleuven.be/geography/projects/14c-palaeolithic), (ii) the Paleoindian Database of the Americas (PIDBA9: 4194 ages; pidba.utk.edu), (iii) the Canadian Archaeological Radiocarbon Database (CARD: 6773 ages; canadianarchaeology.ca), and dates of anatomically modern humans reported in (Auraujo et al, 2017) and (Prates et al, 2020) for Japan and South America respectively.

We gathered a total of 67,643 human mitochondrial Control Region sequences from Genebank (done last on 28 May 2017).

Sampling strategy

Given the large number of archaeological specimens to evaluate, we spatially subdivided America and Eurasia into 2.5 ⍰ 2.5 º grid cells and focussed on obtaining a quality rating for the five oldest records within each grid cell (those records are the most useful in the calculation of the ‘true’ timing of initial human arrival accounting for the Signor-Lipps effect) — see details in Saltre et al, 2015

Data collection

see Research sample

Timing and spatial scale

Our dataset contains a total of 5,977 reliable (after quality rating) specimen ages associated with human presence up to 2020.

We gathered a total of 67,643 human mitochondrial Control Region sequences from Genebank (done last on 28 May 2017).

We limited our study to Eurasia and the Americas

Data exclusions

Since the evaluation of the quality of estimated ages of radiocarbon-dated archaeological specimens is a pre-requisite for any subsequent modelling, inference, and interpretation of records of past life and human impacts (Rodríguez-Rey, M. et al, 2015), we based the quality-rating criteria for these records on Barnosky, & Lindsey (2010), with only dates ranked 13 or higher (out of a possible score of 17) deemed acceptable. We used information pertaining to the method of radiocarbon dating (either standard or accelerator mass spectrometry), the type of archaeological evidence associated with the date (e.g., charcoal, whole bone, bone collagen, bone apatite, wood, hide, hair, peat, organic soil, hydroxyproline, shells), the stratigraphic association between the archaeological evidence and the dated material (i.e., direct or indirect association), as well as the type of material dated to assign each record a numbered rank to assess the reliability of the date. We excluded ages if (i) the author(s) stated that the date was anomalous or (ii) had issues with the extraction protocol, (iii) was/were suspicious of contamination of the sample, or (iv) there was not enough information available to assess reliability. When the ages were not directly identified from human remains, but were instead associated with the age of the layer in which the artefacts were found (i.e., indirect association), we assumed that any material coming from Middle Palaeolithic layers (from 300,000 to 50,000 years ago) was associated with Neanderthals, whereas any material from the Upper Palaeolithic (from 50,000 to 12,000 years ago) was associated with anatomically modern humans (Higham et al, 2014). We disregarded any material in the transition from Middle to Upper Palaeolithic since there are transitional industries whose attribution is controversial. Grounds for excluding dates from controversial South American sites are detailled in Prates et al., 2020. This filtering resulted in a total of 5,977 reliable specimen ages associated with human presence (Supplementary Table S1).

We excluded ancient DNA data and retained 27,506 sequences with reliable geographical locations for further analyses. Mitochondrial haplogroup were classified from Control Region sequences using HaploGrep 2, according to nomenclature provided by PhyloTree mtDNA tree Build 17 (phylotree.org) and we sorted the data by geographical locations.

Reproducibility

Our results are based on a new modelling technique. To ensure the robustness and the reproducibility of our results, we validated our approach against hundreds of scenarios of simulated datasets, and the error map presents a confidence interval around the estimated time of human arrival of < 3000 years for most of the study area.

Randomization

Given that we used a quality-rating approach to vet and select our data, no randomisation procedure was required. We only used a randomisation test to evaluate whether a given environmental variable is a potential driver of the primary human-expansion pathways compared to other trajectories.

Blinding	The blinding procedure was not relevant because each age (fossil or archaeological evidence) was carefully quality-checked to ensure that we used the most reliable dataset

Did the study involve field work? ☐ Yes ☒ No

Reporting for specific materials, systems and methods

We require information from authors about some types of materials, experimental systems and methods used in many studies. Here, indicate whether each material, system or method listed is relevant to your study. If you are not sure if a list item applies to your research, read the appropriate section before selecting a response.

Materials & experimental systems

n/a	Involved in the study
☒	☐ Antibodies
☒	☐ Eukaryotic cell lines
☒	☐ Palaeontology and archaeology
☒	☐ Animals and other organisms
☒	☐ Human research participants
☒	☐ Clinical data
☒	☐ Dual use research of concern

Methods

n/a	Involved in the study
☒	☐ ChIP-seq
☒	☐ Flow cytometry
☒	☐ MRI-based neuroimaging
